# Kinesiology taping and ankle foot orthosis equivalent therapeutic effects on gait function in stroke patients with foot drop: A preliminary study

**DOI:** 10.1097/MD.0000000000034343

**Published:** 2023-07-14

**Authors:** Jong-Bae Choi, Sang-Hoon Lee, Ji-Su Park

**Affiliations:** a Department of Occupational Therapy, Chosun University, Gwangju, Republic of Korea; b Department of Physical Therapy, Busan Paik Hospital, Busan, Republic of Korea; c Research Institute for Korean Medicine, Pusan National University, Yangsan-Si, Republic of Korea.

**Keywords:** ankle foot orthosis, foot drop, gait, kinesiology taping, stroke

## Abstract

An ankle foot orthosis (AFO) is a standard type of orthosis applied to immediately treat foot drop symptoms. Kinesiology taping (KT) is a therapeutic method used in patients with neurological diseases, such as stroke, as well as in patients after orthopedic and sports injuries. This study aimed to compare outcomes of AFO treatment with those of KT to investigate the effect on gait ability in patients with foot drop after stroke. We recruited 18 patients exhibiting foot drop from stroke. Gait ability was assessed under 2 conditions: treatment with KT and that with AFO using the GAITRite system according to the following parameters: cadence, velocity, swing time, stance time, step length, and stride length. As a result, gait ability after treatment with KT and that after treatment with AFO showed no significant differences in cadence (*P* = .851), velocity (*P* = .865), swing time (*P* = .289 and .123), stance time (*P* = .255 and .711), step length (*P* = .955 and .975), and stride length (*P* = .711 and .690) of the affected and less-affected limbs. This study demonstrated that KT and AFO use have similar effects on gait function in patients with foot drop after stroke. Thus, treatment of foot drop with KT may be an alternative in patients for whom AFO use is contraindicated.

## 1. Introduction

Stroke refers to a sudden loss of neurological function due to obstruction of blood flow to the brain caused by ischemia or hemorrhage.^[1]^ Two-thirds of stroke patients have problems with gait after the onset, approximately 30% of stroke patients say that they cannot walk without assistance 6 months after the onset, and only about 70% of stroke patients can safely walk in the community after discharge.^[2]^ Gait refers to moving from one place to another according to the basic needs of an individual. It is one of the most basic daily activities of people. It is an essential human activity that enables us to be productive and participatory members of our community. A decrease in gait function can be considered social isolation due to decreased motivation for social activities and decreased participation in external activities.^[3]^ The greatest sense of loss experienced by patients after stroke is due to a decrease or loss of gait function. Among them, asymmetrical gait is increased owing to foot drop, which is one of the causes of reduced gait function; accordingly, gait velocity is reduced, functional movement is limited, and the risk of falling increases.^[4]^

Foot drop is a common symptom of hemiplegia after stroke and causes weakness of the ankle dorsiflexors due to peroneal nerve paralysis.^[5]^ Gait abnormality occurs from decreased or absent dorsiflexion of the ankle during the swing phase of the gait cycle.^[6]^ Therefore, mitigation of foot drop symptoms after stroke is important for safe and efficient walking. Previous studies have reported the use of ankle foot orthosis (AFO), functional electrical stimulation, partial weight-supported treadmill training, robot-assisted walking therapy, and virtual reality walking training to improve foot drop gait.^[3,4]^ AFO is a standard type of orthosis applied to immediately treat foot drop symptoms.^[7]^ An AFO partly restricts the range of motion of the ankle to alleviate or prevent foot drop symptoms during walking, improving safety or fitted with a rehabilitation for gait training.^[6,8,9]^ Previous studies have shown that an AFO allows for faster walking speed and improved stability by preventing foot drop and thereby maintaining the suitable position of the ankle joint during the swing phase of the gait cycle.^[10,11]^ Therefore, AFOs have been used by patients with stroke and foot drop for several years. Nonetheless, AFO use may be contraindicated in certain patients. As an AFO prevents foot drop by restricting the movement of the ankle during walking, it can cause disuse atrophy and weakness in patients with residual ankle muscle strength after stroke from a lack of volitional effort to use the ankle muscles during walking. This is a consequence of inactivity of the cortex area related with ankle motion and can be explained by the learned nonuse phenomenon theory. In addition, since most AFO are made of plastic, friction with the skin of the soles of the feet can cause side effects such as pain and paresthesia.^[12]^ Therefore, it is important to develop and utilize effective assistive equipment that can overcome the limitations of AFO use to treat foot drop.

Several previous studies have reported a method to replace the standard AFO to treat foot drop. Hwang et al fabricated a dual AFO using thin pierced plastic pieces, straps, and pads.^[13]^ As a result, stability was increased during the stance phase on the affected side and mobility increased during the swing phase on the non-affected side than that in barefoot walking in patients with stroke. Other recent studies have shown that use of the Thera-Band in patients with foot drop after stroke improved walking ability and demonstrated a similar function to that of traditional AFO.^[11,14,15]^ Previous studies, however, have had difficulty applying on their own because of the time-consuming efforts and techniques required to apply the Thera-Band. Therefore, a new treatment method is needed to overcome these shortcomings.

Kinesiology taping (KT) is a therapeutic method used in patients with neurological diseases, such as stroke, as well as in patients after orthopedic and sports injuries.^[16–18]^ This technique not only provides joint stability but also induces immediate activation of the muscles.^[19–21]^ The application of KT to the lower extremities of stroke patients has been reported to positively affect walking ability by reducing pain and stiffness, enhancing muscle activation, and improving balance ability.^[14]^ According to previous studies, KT not only contributes to ankle stability while walking in stroke patients, but is also effective in improving walking ability.^[22,23]^ Recently, Bae et al recently reported a temporary improvement in static balance after applying KT to patients with foot drop after stroke.^[24]^ In addition, Kim et al reported improvements in gait velocity through KT application in chronic stroke patients with foot drop.^[25]^

However, previous studies mainly used evaluation tools such as the Timed up and Go test, 10-meters walk test, 6-minutes’ walk test and balance test, so the effect of KT on various indicators related to walking was unknown. Therefore, it is important to establish a clinical outcome through objective evaluation using quantitative indicators. Complementing the limitations of previous studies, this study was to investigate the effect of KT on the gait ability of patients with foot drop after stroke using an objective quantitative assessment method.

## 2. Materials and methods

### 2.1. Participants

This study enrolled 18 patients with foot drop after stroke from local hospitals in South Korea. The inclusion criteria were as follows: diagnosis with stroke; presence of foot drop symptoms; ability to walk independently or with minimal assistance; modified Ashworth scale was >grade 3; manual muscle testing of the ankle dorsiflexor less than fair grade; and intact lower limb sensory status. The exclusion criteria were as follows: underlying skin disorders; surgery planned for the ankle joint in the next 6 months; exhibiting visuospatial neglect; presence of pain, inflammation, or swelling in the affected leg; bilaterally affected limbs; and presence of anatomical structural anomaly of the foot or ankle. We explained the objectives and requirements of the study to the participants and obtained signed informed consent forms. Ethical approval was obtained from the Seoul Bukbu Hospital Institutional Review Board before conducting the experiment (Seoul April 11, 2019).

### 2.2. Study procedure

The research was conducted in a quiet space where there were no other people, and all risk factors that could lead to falls, such as obstacles, were removed from the floor. The patient was evaluated to be in a stable state after receiving enough rest. This study investigated the effect of KT and AFO application interventions on gait ability. Patients walked under 2 conditions and were evaluated by a physical therapist. The evaluation order of the 2 conditions was determined using an envelope randomization. After each walking condition, we allowed a break of 5 minutes. Before the test, patients wore the KT and AFO and practiced 2 to 3 round trips on the gait mat. The average of 3 gait values was analyzed. However, if data extraction was not possible due to sensor error of the gait mat during walking, we repeated the measurement.

### 2.3. KT application

**Figure 1.**
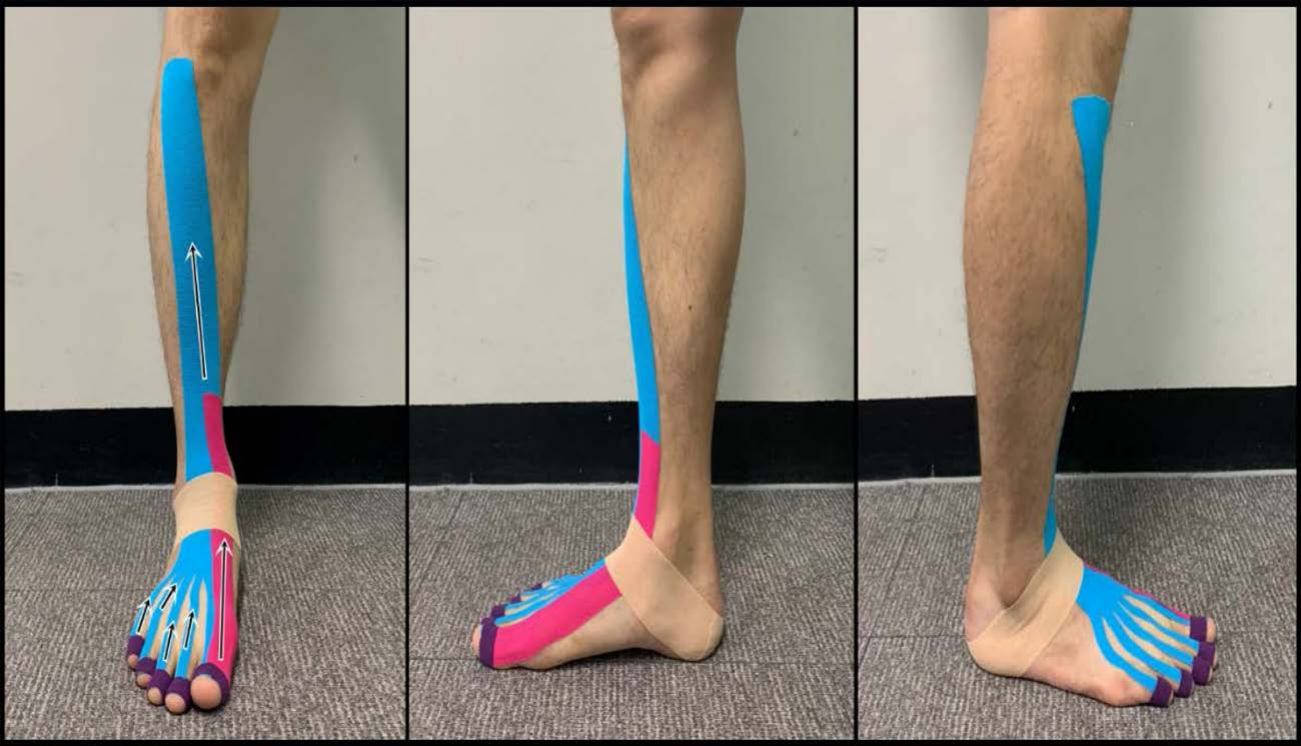
The application of designed kinesiology taping. Left: frontal view of KT; center: inner lateral view of KT; right: outer lateral view.

The kinesiology tape (BB Tape, WETAPE Inc., Seoul, Korea) was applied with approximately 50% stretch by a skilled occupational therapist who had a KT certification and more than 8 years of experience. KT was applied in 4 steps as follows. First, the therapist maintained the toe extension and dorsiflexion of the patient’s ankle joint. Second, KT was attached vertically to the tibialis anterior muscle (TAm) in the anterior leg area from the toe interphalangeal joint to the dorsal surface and ankle joint of the foot. Third, the clinician wrapped the interphalangeal joint of the toe for firm attachment of the tape. Fourth, they wrapped the ankle joint (talus to the calcaneus) for firm attachment of the tape (Fig. 1).

### 2.4. AFO application

A commercially available, partially open-type AFO (UD-flex, ADVANFIT Inc., Kumamoto, Japan) was used in this study. All participants were fitted with AFO by skilled occupational therapist.

### 2.5. Outcome measurements

**Figure 2.**
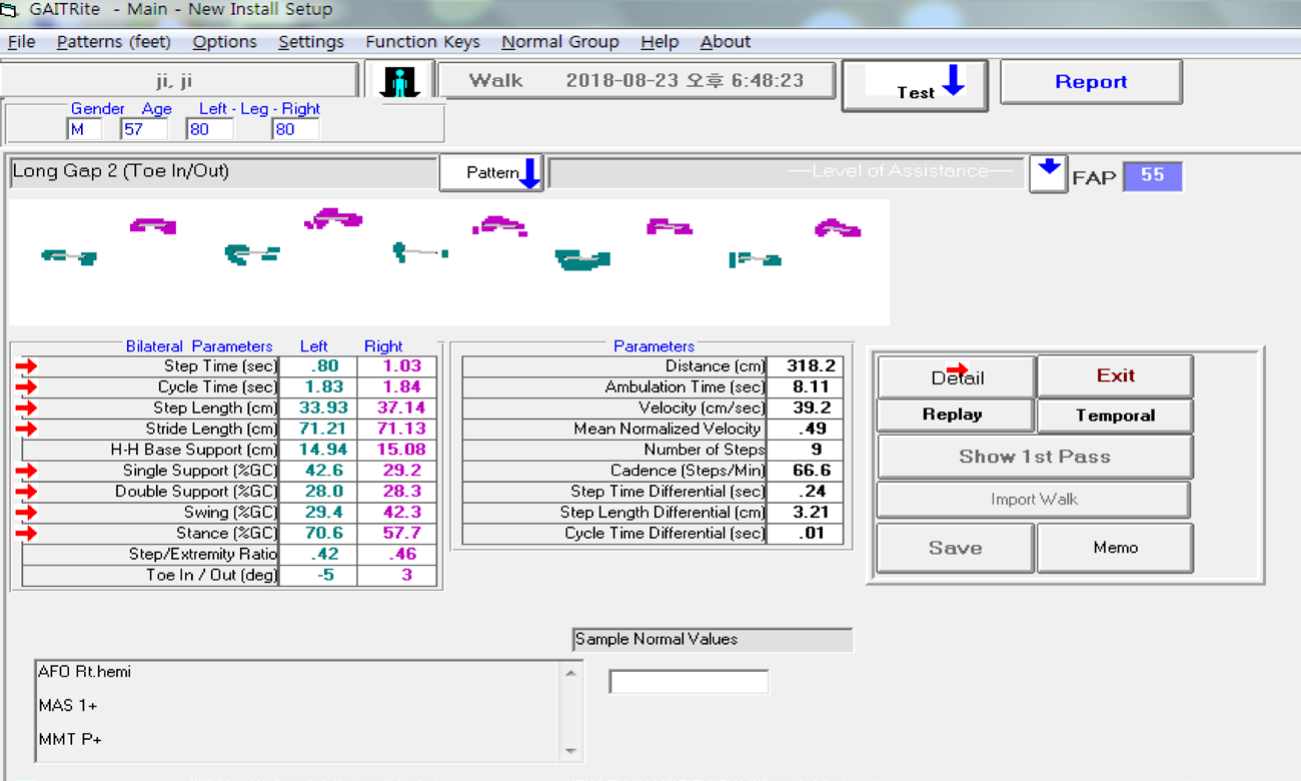
The scene of GAITRite software. Six parameters were employed to estimate features of walking ability.

In this study, The GAITRite system was used to quantitatively evaluate the walking ability of participants. This evaluation equipment automatically analyzes temporal and spatial gait parameters during walking. The sensor pads of the GAITRite were covered with a rolled-up carpet, which provided an active measurement area activated by mechanical pressure from foot contact to the mat. Data from the activated sensors were collected on a computer at a sampling rate of 80 Hz, and the footsteps were identified. The parameters were calculated automatically (Fig. 2).^[26]^ The validity and reliability of the device have been previously established.^[27]^

### 2.6. Data analysis

In this study, Wilcoxon signed rank test was used to compare the degree of gait in KT and AFO states. We used SPSS software (ver. 18.0 for Windows; SPSS, Chicago, IL). The level of statistical significance was set at *P* < .05.

## 3. Results

### 3.1. General characteristics of participants

In this study, 18 patients were recruited and completed the experiments. Data from all 18 participants were thus included in the analysis. Table 1 shows the general characteristics of the participants.

**Table 1:** Demographic characteristics of the subjects.

Subject	Gender	Age (years)	Stroke type	Paratic limb	Since onset (weeks)	Height (cm)	Weight (kg)
1	Man	55	Hemorrhage	Right	16	175	75
2	Man	60	Hemorrhage	Right	21	171	73
3	Man	61	Hemorrhage	Left	18	168	67
4	Man	59	Infarction	Right	22	168	70
5	Man	58	Infarction	Right	15	165	64
6	Man	53	Hemorrhage	Left	20	168	68
7	Man	67	Hemorrhage	Left	18	162	60
8	Man	60	Infarction	Left	24	174	71
9	Man	63	Hemorrhage	Right	16	160	62
10	Man	47	Infarction	Left	28	179	77
11	Woman	58	Hemorrhage	Right	20	156	51
12	Woman	62	Hemorrhage	Right	15	152	52
13	Woman	69	Infarction	Left	29	155	50
14	Woman	52	Infarction	Left	10	161	55
15	Woman	48	Hemorrhage	Right	31	164	56
16	Woman	66	Hemorrhage	Right	16	155	49
17	Woman	71	Hemorrhage	Right	22	153	50
18	Woman	58	Infarction	Left	33	162	54

### 3.2. Assessment of gait

We found no significant difference in cadence (*P* = .851) and velocity (*P* = .865). In addition, there was no significant difference in swing time (*P* = .289 and .123), stance time (*P* = .255 and .711), step length (*P* = .955 and .975), and stride length (*P* = .711 and .690) of the affected limbs (Table 2).

**Table 2:** Comparison of gait parameters with KT and AFO in stroke patients.

	KT condition	AFO condition	*t*	*P*
Cadence (steps/min)	57.42 ± 11.60	58.43 ± 11.23	−.379	.851
Velocity (s)	26.64 ± 7.37	26.41 ± 5.57	.119	.865
Swing time (s)				
Affected side	0.71 ± 0.16	0.69 ± 0.14	.971	.289
Less-affected side	0.44 ± 0.09	0.42 ± 0.07	1.057	.123
Stance time (s)				
Affected side	1.62 ± 0.10	1.61 ± 0.11	.654	.255
Less-affected side	2.23 ± 0.34	2.24 ± 0.31	−.288	.711
Step length (cm)				
Affected side	24.73 ± 4.93	24.68 ± 5.23	.027	.955
Less-affected side	26.27 ± 5.52	26.77 ± 4.61	−.291	.975
Stride length (cm)				
Affected side	49.89 ± 5.59	49.00 ± 8.01	.352	.711
Less-affected side	51.66 ± 7.21	53.62 ± 7.78	−.867	.690

Values are mean ± SD.

AFO = ankle foot orthosis, KT = kinesiology taping.

### 3.3. Report of side effects related to KT

There were no reported adverse events throughout this study. However, after completion of the experiment, 6 out of 18 patients reported discomfort due to adhesion when removing KT from the leg. The AFO, on the other hand, did not cause any discomfort.

## 4. Discussion

Foot drop has a negative effect on daily life because it causes gait disturbances. Therefore, the prevention and rehabilitation of foot drop in stroke patients are very important in clinical practice.^[28]^ KT is a rehabilitation method that is commonly used in gait rehabilitation and is a safe and convenient method that can be applied to the lower extremities including toes, ankles, and legs.^[18,29–31]^ Because KT has the characteristics of adhesion and elongation, it has the advantage of being easily attached to and removed from the skin surface, and it can induce physical changes in joints and soft tissues through elongation.^[32]^ Therefore, it is possible to reduce foot drop symptoms during walking by using these characteristics of KT. This study performed a spatiotemporal evaluation to investigate the effect of KT and AFO use on walking ability. This study applied KT and AFO to 18 patients with hemiplegia after stroke and found no significant difference in all parameters, namely cadence, velocity, swing time, stance time, step length, and stride length. These results suggest that AFO and KT have similar effects on walking ability, which can be explained as follows. AFO use alleviates foot drop symptoms of the ankle during walking through the principle of three-point pressure. Conversely, KT prevents foot drop symptoms based on a different principle. Since KT is characterized by adhesion and elasticity, it can partially limit the range of motion of the joint due to the attachment of the tape to the skin.^[33]^ In this study, the ankle joint was passively attached to the anterior leg via the dorsal surface of the ankle joint at the toe joint. Thus, the elastic mechanism of the taping was utilized, rendering the physical external force of KT helpful in improving walking ability by maintaining a dorsiflexed state of the ankle joint during walking. Additionally, foot drop symptoms are treated via the effects of muscle facilitation through KT. KT is known to facilitate or activate a small but immediate increase in muscle strength by producing a concentric pull on the fascia, which may stimulate an increase in muscle contraction.^[34]^ In this study, KT was applied from the toe to leg and the target muscles were the TAm, extensor digitorum longus, and extensor hallucis longus. The TAm is a muscle located on the front of the leg that enables dorsiflexion of the ankle joint during walking. KT may have a positive effect on the ankle dorsiflexion during walking by inducing more muscle activation in the TAm. In addition, KT applied to these muscles can help with toe off in the stance phase of gait through temporary muscle activation, wherein the improvement of gait ability can be expected by the influence of ground reaction force. This also acts later in the gait cycle to enable the foot clear the ground during the swing phase.^[35]^ This study demonstrated that KT has an effect similar to that of AFO use on walking ability in patients with foot drop after stroke. AFO causes some discomfort when walking because it is relatively hard and thick owing to the characteristics of the material. However, since the KT material is very soft and thin, not only does it feel less heterogeneous during walking, but there is almost no difference in height, with respect to the opposite leg, in a standing state. Therefore, this intervention can be an alternative to AFO for foot drop patients. In particular, no side effects were reported in any of the participants during the experiment using KT in this study. However, many patients had some discomfort during the process of removing the KT owing to hair on their leg. We suggest that the following matters should be considered when applying KT. In particular, factors such as KT type, application strength, and design should be considered and applied. First, KT’s elongation force. If the elongation force of the KT is too low, it may not be able to generate a physical force that can support the ankle joint.^[9]^ However, if the stretching force of KT is too high, it is important to apply an appropriate stretching force because it causes excessive dorsiflexion of the ankle during walking, which obstructs walking.^[36]^ Therefore, it is necessary to adjust tension in consideration of the patient’s physical characteristics or walking ability. Second, depending on the application design of KT, gait or application compliance may be affected.^[20]^ In this study, the tape was pulled from the tip of the toe, passed through the dorsal surface of the ankle, and attached to the leg. However, depending on the patient’s walking ability, it is possible to design it in a shorter or figure 8 form and try to attach it. Therefore, it is necessary to verify its clinical effects by designing it in various forms. Finally, the type of tape. KT is primarily divided into elastic and inelastic types. The characteristic of an elastic tape is its tensile strength. That is, tension can be provided differently by increasing the length as desired by the user. While non-stretchable tape has relatively low elasticity, it is advantageous in supporting or fixing the structure.^[37]^ Therefore, it is important to consider each of these features appropriately when applying KT for swallowing rehabilitation. Therefore, it is clinically necessary to consider various factors such as KT type, application strength, and design for gait rehabilitation of patients with foot drop after stroke.

### 4.1. Study limitations

This study had some limitations that need to be taken into account while interpreting the data. First, the number of participants was low; hence, the results cannot be generalized. Second, this study could not be blinded to the assessor because of restricted staff availability. Third, since other general rehabilitation treatment was performed in the hospital, it is thought that it may affect the treatment result. Finally, in this study, KT was applied with approximately 50% stretch, but it is somewhat difficult to quantitatively measure this. In future research, these limitations need to be overcome, and various designs of KT need to be developed with a demonstration of their effectiveness. In addition, if KT and other interventions with proven effectiveness are applied in parallel, it is expected to contribute a lot to the gait rehabilitation of stroke patients through positive synergistic effects.

## 5. Conclusions

We demonstrated that KT and AFO use have a similar effect on walking ability in patients with foot drop after stroke. Therefore, KT is proposed as an alternative to AFO use to improve walking ability in stroke patients where AFO use may be contraindicated.

## Author contributions

**Conceptualization:** Sang-Hoon Lee.

**Formal analysis:** Jong-Bae Choi, Sang-Hoon Lee.

**Investigation:** Jong-Bae Choi, Ji-Su Park.

**Methodology:** Jong-Bae Choi.

**Resources:** Jong-Bae Choi, Ji-Su Park.

**Software:** Ji-Su Park.

**Supervision:** Sang-Hoon Lee, Ji-Su Park.

**Validation:** Jong-Bae Choi.

**Visualization:** Sang-Hoon Lee.

**Writing – review & editing:** Ji-Su Park.

## References

[1] Hara Y. Brain plasticity and rehabilitation in stroke patients. J Nippon Med Sch. 2015;82:4–13. 25797869 10.1272/jnms.82.4

[2] Sotomayor-Sobrino MA Ochoa-Aguilar A Mendez-Cuesta LA. Neuroimmunological interactions in stroke [Interacciones neuroinmunologicas en el ictus]. Neurologia (Engl Ed). 2019;34:326–35. 27776957 10.1016/j.nrl.2016.08.003

[3] Sheng Y Kan S Wen Z. Effect of kinesio taping on the walking ability of patients with foot drop after stroke. Evid Based Complement Alternat Med. 2019;2019:2459852. 31223327 10.1155/2019/2459852 PMC6541939

[4] Park D Lee KS. Effects of talus stabilization taping versus ankle kinesio taping in patients with chronic stroke: a randomized controlled trial. J Exerc Rehabil. 2019;15:775–80. 31938698 10.12965/jer.1938642.321 PMC6944872

[5] Prenton S Kenney LP Stapleton C. Feasibility study of a take-home array-based functional electrical stimulation system with automated setup for current functional electrical stimulation users with foot-drop. Arch Phys Med Rehabil. 2014;95:1870–7. 24845222 10.1016/j.apmr.2014.04.027

[6] Karniel N Raveh E Schwartz I. Functional electrical stimulation compared with ankle-foot orthosis in subacute post stroke patients with foot drop: a pilot study. Assist Technol. 2021;33:9–16. 30945999 10.1080/10400435.2019.1579269

[7] Prenton S Hollands KL Kenney LPJ. Functional electrical stimulation and ankle foot orthoses provide equivalent therapeutic effects on foot drop: a meta-analysis providing direction for future research. J Rehabil Med. 2018;50:129–39. 29227525 10.2340/16501977-2289

[8] Park JS Lee SH Yoo WG. Immediate effect of a wearable foot drop stimulator to prevent foot drop on the gait ability of patients with hemiplegia after stroke. Assist Technol. 2021;33:313–7. 31311426 10.1080/10400435.2019.1634658

[9] de Paula GV da Silva TR de Souza JT. Effect of ankle-foot orthosis on functional mobility and dynamic balance of patients after stroke: study protocol for a randomized controlled clinical trial. Medicine (Baltim). 2019;98:e17317. 10.1097/MD.0000000000017317 PMC6775434 31574862

[10] Gatti MA Freixes O Fernandez SA. Effects of ankle foot orthosis in stiff knee gait in adults with hemiplegia. J Biomech. 2012;45:2658–61. 22980576 10.1016/j.jbiomech.2012.08.015

[11] Song S Park J Song G. Usability of the Thera-Band® to improve foot drop in stroke survivors. NeuroRehabilitation. 2018;42:505–10. 29660954 10.3233/NRE-172338

[12] Vasiliauskaite E Ielapi A De Beule M. A study on the efficacy of AFO stiffness prescriptions. Disabil Rehabil Assist Technol. 2021;16:27–39. 31226898 10.1080/17483107.2019.1629114

[13] Hwang YI An DH Yoo WG. Effects of the dual AFO on gait parameters in stroke patients. NeuroRehabilitation. 2012;31:387–93. 23232162 10.3233/NRE-2012-00808

[14] Hwang YI Yoo WG An DH. Effects of the Elastic Walking Band on gait in stroke patients. NeuroRehabilitation. 2013;32:317–22. 23535794 10.3233/NRE-130850

[15] Hwang YI Yoo WG An DH. The effect of an AFO-shaped elastic band on drop-foot gait in patients with central neurological lesions. NeuroRehabilitation. 2013;32:377–83. 23535802 10.3233/NRE-130858

[16] Yang L Yang J He C. The effect of kinesiology taping on the hemiplegic shoulder pain: a randomized controlled trial. J Healthc Eng. 2018;2018:8346432. 30651946 10.1155/2018/8346432 PMC6311752

[17] Park JS Yoon T Lee SH. Immediate effects of kinesiology tape on the pain and gait function in older adults with knee osteoarthritis. Medicine (Baltim). 2019;98:e17880. 10.1097/MD.0000000000017880 PMC6855641 31702659

[18] Park D Bae Y. Proprioceptive neuromuscular facilitation kinesio taping improves range of motion of ankle dorsiflexion and balance ability in chronic stroke patients. Healthcare (Basel). 2021;9:1426. 34828473 10.3390/healthcare9111426 PMC8619064

[19] Yun HG Lee JH Choi IR. Effects of kinesiology taping on shoulder posture and peak torque in junior baseball players with rounded shoulder posture: a pilot study. Life (Basel). 2020;10:139. 32781512 10.3390/life10080139 PMC7459854

[20] Huang CY Hsieh TH Lu SC. Effect of the kinesio tape to muscle activity and vertical jump performance in healthy inactive people. Biomed Eng Online. 2011;10:70. 21831321 10.1186/1475-925X-10-70 PMC3174125

[21] Huang TS Ou HL Lin JJ. Effects of trapezius kinesio taping on scapular kinematics and associated muscular activation in subjects with scapular dyskinesis. J Hand Ther. 2019;32:345–52. 29196161 10.1016/j.jht.2017.10.012

[22] Park D Lee JH Kang TW. Immediate effects of talus-stabilizing taping on balance and gait parameters in patients with chronic stroke: a cross-sectional study. Top Stroke Rehabil. 2018;25:417–23. 29717946 10.1080/10749357.2018.1466972

[23] Wang M Pei ZW Xiong BD. Use of kinesio taping in lower-extremity rehabilitation of post-stroke patients: a systematic review and meta-analysis. Complement Ther Clin Pract. 2019;35:22–32. 31003662 10.1016/j.ctcp.2019.01.008

[24] Bae YH Kim HG Min KS. Effects of lower-leg kinesiology taping on balance ability in stroke patients with foot drop. Evid Based Complement Alternat Med. 2015;2015:125629. 26579200 10.1155/2015/125629 PMC4633546

[25] Kim BJ Lee JH Kim CT. Effects of ankle balance taping with kinesiology tape for a patient with chronic ankle instability. J Phys Ther Sci. 2015;27:2405–6. 26311206 10.1589/jpts.27.2405 PMC4540890

[26] Webster KE Wittwer JE Feller JA. Validity of the GAITRite walkway system for the measurement of averaged and individual step parameters of gait. Gait Posture. 2005;22:317–21. 16274913 10.1016/j.gaitpost.2004.10.005

[27] Bilney B Morris M Webster K. Concurrent related validity of the GAITRite walkway system for quantification of the spatial and temporal parameters of gait. Gait Posture. 2003;17:68–74. 12535728 10.1016/s0966-6362(02)00053-x

[28] Alnajjar F Zaier R Khalid S. Trends and technologies in rehabilitation of foot drop: a systematic review. Expert Rev Med Devices. 2021;18:31–46. 33249938 10.1080/17434440.2021.1857729

[29] Shin YJ Lee JH Choe YW. Immediate effects of ankle eversion taping on gait ability of chronic stroke patients. J Bodyw Mov Ther. 2019;23:671–7. 31563387 10.1016/j.jbmt.2018.06.008

[30] Choi HS Lee JH. Immediate effect of balance taping using kinesiology tape on dynamic and static balance after ankle muscle fatigue. Healthcare (Basel). 2020;8:162. 32526892 10.3390/healthcare8020162 PMC7348943

[31] Khalili SM Barati AH Oliveira R. Effect of combined balance exercises and kinesio taping on balance, postural stability, and severity of ankle instability in female athletes with functional ankle instability. Life (Basel). 2022;12:178. 35207466 10.3390/life12020178 PMC8879431

[32] Lee JH Choi IR Choi HS. Immediate effects of ankle-foot orthosis using wire on static balance of patients with stroke with foot drop: a cross-over study. Healthcare (Basel). 2020;8:116. 32354201 10.3390/healthcare8020116 PMC7349351

[33] Park JS Jung YJ Kim HH. A novel method using kinesiology taping for the activation of suprahyoid muscles in healthy adults: a preliminary research. Dysphagia. 2020;35:636–42. 31620860 10.1007/s00455-019-10071-4

[34] Kaya Mutlu E Mustafaoglu R Birinci T. Does kinesio taping of the knee improve pain and functionality in patients with knee osteoarthritis? a randomized controlled clinical trial. Am J Phys Med Rehabil. 2017;96:25–33. 27149590 10.1097/PHM.0000000000000520

[35] Koseoglu BF Dogan A Tatli HU. Can kinesio tape be used as an ankle training method in the rehabilitation of the stroke patients? Complement Ther Clin Pract. 2017;27:46–51. 28438279 10.1016/j.ctcp.2017.03.002

[36] Huang YC Chang KH Liou TH. Effects of kinesio taping for stroke patients with hemiplegic shoulder pain: a double-blind, randomized, placebo-controlled study. J Rehabil Med. 2017;49:208–15. 28233009 10.2340/16501977-2197

[37] Hu Y Zhong D Xiao Q. Kinesio taping for balance function after stroke: a systematic review and meta-analysis. Evid Based Complement Alternat Med. 2019;2019:8470235. 31379969 10.1155/2019/8470235 PMC6662277

